# The Acceptability and Impact of the Xploro Digital Therapeutic Platform to Inform and Prepare Children for Planned Procedures in a Hospital: Before and After Evaluation Study

**DOI:** 10.2196/17367

**Published:** 2020-08-11

**Authors:** Lucy Bray, Ashley Sharpe, Phillip Gichuru, Peter-Marc Fortune, Lucy Blake, Victoria Appleton

**Affiliations:** 1 Faculty of Health, Social Care and Medicine Edge Hill University Ormskirk United Kingdom; 2 Royal Manchester Children's Hospital Manchester United Kingdom

**Keywords:** health literacy, augmented reality, children, procedure, health, artificial intelligence

## Abstract

**Background:**

There is increasing interest in finding novel approaches to improve the preparation of children for hospital procedures such as surgery, x-rays, and blood tests. Well-prepared and informed children have better outcomes (less procedural anxiety and higher satisfaction). A digital therapeutic (DTx) platform (Xploro) was developed with children to provide health information through gamification, serious games, a chatbot, and an augmented reality avatar.

**Objective:**

This before and after evaluation study aims to assess the acceptability of the Xploro DTx and examine its impact on children and their parent’s procedural knowledge, procedural anxiety, and reported experiences when attending a hospital for a planned procedure.

**Methods:**

We used a mixed methods design with quantitative measures and qualitative data collected sequentially from a group of children who received standard hospital information (before group) and a group of children who received the DTx intervention (after group). Participants were children aged between 8 and 14 years and their parents who attended a hospital for a planned clinical procedure at a children’s hospital in North West England. Children and their parents completed self-report measures (perceived knowledge, procedural anxiety, procedural satisfaction, and procedural involvement) at baseline, preprocedure, and postprocedure.

**Results:**

A total of 80 children (n=40 standard care group and n=40 intervention group) and their parents participated in the study; the children were aged between 8 and 14 years (average 10.4, SD 2.27 years) and were attending a hospital for a range of procedures. The children in the intervention group reported significantly lower levels of procedural anxiety before the procedure than those in the standard group (two-tailed *t*_63.64_=2.740; *P*=.008). The children in the intervention group also felt more involved in their procedure than those in the standard group (*t*_75_=−2.238; *P*=.03). The children in the intervention group also reported significantly higher levels of perceived procedural knowledge preprocedure (*t*_59.98_=−4.892; *P*=.001) than those in the standard group. As for parents, those with access to the Xploro intervention reported significantly lower levels of procedural anxiety preprocedure than those who did not (*t*_68.51_=1.985; *P*=.05). During the semistructured *write and tell* interviews, children stated that they enjoyed using the intervention, it was fun and easy to use, and they felt that it had positively influenced their experiences of coming to the hospital for a procedure.

**Conclusions:**

This study has shown that the DTx platform, Xploro, has a positive impact on children attending a hospital for a procedure by reducing levels of procedural anxiety. The children and parents in the intervention group described Xploro as improving their experiences and being easy and fun to use.

## Introduction

Children can find visiting a hospital to be a stressful and disorientating experience. Research shows that children can experience high levels of anxiety when attending a hospital for procedures [1], normally due to fear of the unknown, medical examinations, pain, separation from parents, uncertainty, and loss of control [2,3]. These fears and worries can result from poor preparation and information [4,5], and can result in children becoming distressed and uncooperative during a clinical procedure [6]. Anxiety and distress linked to undergoing a procedure can be long-lasting and have implications on children’s subsequent health care encounters and long-term health outcomes [7]. Children’s distress and noncooperation during procedures can also result in longer appointments, delays to appointments, and referrals to psychological services, all of which have cost implications for health service providers. Children and their parents identify an unmet need for information about hospital procedures and interventions [8,9] and that such information would be valuable to help them know what to expect and how best to prepare themselves for a procedure [10].

Traditional forms of preparation for children include leaflets and books [11], and while these have been shown to have some benefit, computer- and app-based interventions have been highlighted as being best placed to deliver preparation information for children coming to the hospital for a planned procedure [12]. There is evidence that computer- and app-based interventions are helpful in educating and preparing children for health experiences, but to date these have focused predominantly on admissions for surgery [13-16] or one particular context (eg, radiology [17]). Although these interventions are valuable, children may encounter numerous clinical procedures, health care professionals, and environments while visiting the hospital. There is a need to develop and robustly evaluate interventions that address the more common interactions and procedures that children encounter within hospitals. Therefore, it was expected that an accessible and child-centered intervention to familiarize and inform children about a broad range of experiences, environments, and health professionals would increase value and improve children’s procedural health literacy and experiences.

Health literacy refers to a person’s ability to access and gain information, understand this information, and use it to communicate and be involved in making health choices and decisions [18,19]. In relation to the Xploro intervention, we proposed that a child’s health literacy would be enhanced by accessing meaningful information (knowledge) through the digital therapeutic (DTx) platform, which they can understand and use to familiarize and prepare themselves for their hospital visits (reduced procedural anxiety, improved experiences, and increased involvement in their procedure). The Xploro intervention has been developed with children based on an *information-pull* design. This design acknowledges that children learn and gain knowledge optimally by actively accessing information and constructing their own understandings through engaging with multiple elements (gamification, augmented reality, serious games, and a chatbot) to influence multiple aspects of a procedure (anxiety, knowledge, involvement, and satisfaction). There is currently a lack of robust research evaluating the use and impact of platforms such as Xploro with children attending a hospital.

This before and after study aims to assess the acceptability of Xploro DTx and examine its impact on children and their parents’ health literacy, perceptions of procedural knowledge, procedural anxiety, procedural involvement, procedural satisfaction, and reported experiences when attending a hospital for a planned procedure.

## Methods

### Study Design

The study was designed as a before and after evaluation study comprising 2 separate groups of children. Data were collected sequentially from a group of children who received standard hospital information (before group) and from a group of children who received the DTx intervention (after group). As the DTx platform can be considered a complex intervention (multiple interacting components with multiple aims), we conducted a before and after study design to enable us to ascertain whether the intervention was acceptable and accessible to children and their parents within a health care setting and evaluate whether the outcomes were favorable [20]. This study would help inform whether further research to assess the effectiveness of the intervention in a larger study could and should be done.

The study used a mixed methods design consisting of structured quantitative measures and qualitative interviews. Self-completion questionnaire booklets were completed separately by children aged 8 to 14 years and their parent or carer at 3 time points: baseline (3 to 5 days before attending a hospital for the procedure), before the procedure (on arrival at the hospital), and after their procedure (within 5-10 min after completing their procedure). The questionnaire booklets collected self-report data on procedural anxiety, perceptions of knowledge, involvement, and satisfaction. The design of the questionnaire booklet was informed by consultations with 10 children and 4 parents to ensure that the directions, language, and measures were easily understood. The children, during this consultation, highlighted that the use of multiple measures or lengthy questionnaires may add to a child’s anxiety when attending a hospital for a procedure, so the study was designed to ensure that participant burden was kept to a minimum.

Short qualitative interviews, focused around a *write and tell* [21] activity sheet, were conducted with children and their parents after the procedure. The interviews explored what children and parents perceived went well during the procedure and what might have made it better. For the children in the intervention group, the interviews also sought their opinions and experiences of using the DTx platform (Xploro) and aimed to gain insight into how children used the intervention within the context of a hospital visit and how it impacted their procedural experiences. The collection of qualitative data from all the children and their parents involved in the study was in recognition of the value of these data as part of an evaluation of a complex intervention [22].

### Participants and Recruitment

Participants were children undergoing a planned procedure in a children’s hospital in North West England and their attending parents. Children were recruited using convenience sampling for the standard care group between September 2018 and January 2019 and the intervention group between January and June 2019. Eligible children were those aged between 8 and 14 years who were to attend a hospital for a planned clinical procedure without a moderate or severe cognitive impairment or a referral to psychological services for procedural anxiety. The age of the children recruited for the study was determined by the target population for the Xploro intervention. Researchers positioned themselves within outpatient and inpatient departments on different days of the week and worked with clinical teams to identify children who were due to return for a clinical procedure within the next few weeks. Eligibility was determined by the clinical team, who initially approached the family with information about the study.

### Study Intervention

**Figure 1:**
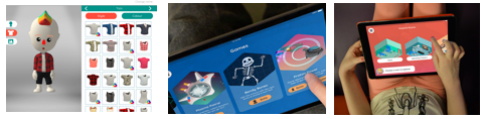
Different components of the Xploro Digital Therapeutic (DTx) platform.

Xploro is a DTx platform that uses augmented reality, gameplay, and artificial intelligence to deliver health information to children (Figure 1). The intervention was developed in response to the personal experience of the founder to a lack of engaging information for children attending a hospital for procedures and treatments. The DTx platform provides information about health environments (wards and operating theaters), key health staff, and hospital equipment. The DTx platform includes an avatar that children can customize and acts as a guide and chatbot. The DTx platform also includes several serious games with health themes. The DTx platform includes information about the procedure, environment and staff, and information on sensory aspects of a procedure (what a child may feel or experience) as well as information to help a child build‑coping strategies [9].

Xploro development has been informed by a user-centered design or person-based approach [23]. Children (n=105), health professionals (n=19), and parents (n=27) were involved in a previous qualitative study to inform the content (chatbot questions and answers, games, and language used), navigation (working through different elements or easily locating the procedure of relevance to them), and access to the DTx app (parents as gatekeepers and importance of timing of procedural information) [9]. This previous work was conducted in acknowledgment of the importance of qualitative research in informing the development of DTx interventions to ensure that they are engaging, acceptable, and effective [24] and that an investment in developmental work is essential before the formal evaluation of complex interventions [20]. Xploro is designed to supplement, not replace, normal forms of information provision and communication between children and health professionals. For this study, Xploro was accessed by children in the intervention group on a preloaded iPad delivered to each family at least 3 days before the planned procedure.

### Measures

**Figure 2:**
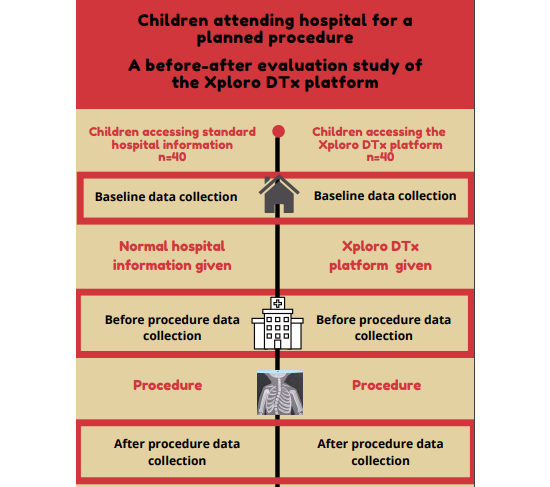
Flow diagram of the study processes.

Self-report questionnaires were completed by children and their parents at baseline (3 to 5 days before the planned procedure), before the procedure (within the hospital immediately before the procedure), and after the procedure (within the hospital and up to 10 min after the procedure was completed; Figure 2; Table 1).

Children and parents’ self-reported ratings were collected for their procedural (state) anxiety (10-point visual analog scale [VAS]), trait anxiety (10-point VAS), procedural knowledge levels (10-point VAS), procedural satisfaction (10-point VAS), and procedural involvement (5-point Likert scale). These were single-item scales.

**Table 1:** Measures used in the study.

Child measures		Parent measures	Points of measurement
**Trait anxiety measure**			
	“How you would usually feel at home?” 0 (completely relaxed and calm) to 10 (completely worried and anxious; adapted from Kleiber and McCarthy, 2006 [25])	“How worried or anxious are you normally about things?” 0 (not at all anxious or worried) to 10 (very anxious or worried) Self-developed	Baseline
**Procedural anxiety measure**			
	“How do you feel now?” 0 (completely relaxed and calm) to 10 (completely worried and anxious; adapted from Kleiber and McCarthy, 2006 [25])	“How anxious or worried are you about your child’s procedure?” 0 (not at all anxious or worried) to 10 (very anxious or worried) Self-developed	Baseline Before the procedure
**Perception of procedural knowledge measure**			
	“How much you know or understand about what will happen to you today at the hospital?” 0 (know nothing) to 10 (know everything) Self-developed	“How much do you know about your child’s procedure?” 0 (know nothing) to 10 (know everything) Self-developed	Baseline Before the procedure
**Procedural satisfaction measure**			
	“How satisfied or happy you are with what happened today?” 0 (not at all happy/satisfied) to 10 (completely happy/satisfied) Self-developed	“Overall how satisfied were you with your child’s procedure today?” 0 (not at all satisfied) to 10 (completely satisfied; adapted from Spencer and Franck, 2005 [26])	After the procedure
**Procedural involvement measure**			
	“Were you involved, as much as you wanted to be, in decisions about your procedure?” 0: No 1: Yes, sort of 2: Yes, definitely Children could also respond: “I did not want or need to be involved and these responses were not included in the analyses”; adapted from Toomey et al, 2015 [27])	N/A^a^	After the procedure
**Intervention engagement measure**			
	“Which parts of the app did you look at /go through?” (Tick against a list of the different elements) “Did you like this part?” (yes/no)	N/A^a^	After the procedure

^a^N/A: not applicable.

### Ethics Approval

Ethics approval was received from the authors’ institution (FOH 194) and the Health Research Authority (18/WA/0277).

### Statistical Analysis

Independent *t* tests were conducted to compare parents and children’s scores on the following measures between the 2 groups (those using the DTx platform intervention and those using standard procedural information when attending a hospital for a planned procedure): trait anxiety, procedural anxiety, perception of procedural knowledge and procedural satisfaction.

Where significant differences were found between the standard and intervention groups, two-tailed paired *t* tests were performed to determine whether there were any differences in parents and children’s scores from baseline to before the procedure. To examine those who benefited the most from Xploro, the following exploratory group comparisons were conducted: (1) those who had invasive procedures versus those who had noninvasive procedures and (2) children who had 3 or more visits to the hospital and those who had fewer than 3 visits to the hospital.

Descriptive statistics have been presented regarding engagement with the intervention.

All analyses were conducted using SPSS version 25 (IBM Corp), and *P*=.04 was considered statistically significant.

### Qualitative Analysis

The text responses of children and their parents were inductively analyzed by 2 researchers using content analysis processes [28], where the responses were coded and then organized into broad themes.

## Results

### Participant Characteristics

Of the 80 children and parents eligible to participate, all were successfully recruited, and all of them completed the baseline, preprocedural, and postprocedural data collection processes. Participants were aged between 8 and 14 years (average 10.5 years for the standard group and 12 years for the intervention group) and were attending a hospital for a range of procedures. The characteristics between the 2 groups were similar, for example, age, gender, previous hospital experience, and parental education (Table 2).

**Table 2:** Characteristics of the participants.

Characteristics		Usual hospital information (before) group, n (%)		Xploro digital therapeutic platform (intervention) group, n (%)	
**Child’s age, (years)**					
	8-10		16 (40)		20 (50)
	11-14		24 (60)		20 (50)
**Gender**					
	Male		20 (50)		16 (40)
	Female		20 (50)		24 (60)
**Type of procedure**					
	Noninvasive (x-ray and ultrasound)		14 (35)		9 (23)
	Invasive (surgery cannulation and blood tests)		26 (65)		31 (78)
**Previous hospital procedure**					
	<3 previous hospital experiences		22 (55)		26 (65)
	>3 previous hospital experiences		18 (45)		14 (35)
**Parent educational level**					
	Primary		1 (3)		0 (0)
	Secondary		22 (55)		27 (68)
	Graduate		12 (30)		11 (28)
	Postgraduate		4 (10)		2 (5)

### Parents and Children’s Levels of Trait Anxiety

At baseline, levels of general anxiety were similar for both parents and children in the 2 groups. Specifically, parents’ levels of anxiety did not differ between the standard (mean 2.15, SD 0.770) and intervention groups (mean 2.10, SD 0.709; *t*_78_=0.302; *P*=.76). Similarly, children’s levels of trait anxiety did not differ between the standard (mean 2.35, SD 0.700) and intervention groups (mean 2.38, SD 0.49; *t*_69.85_=−1.85; *P*=.85).

### Feeling Less Worried: Reducing Procedural Anxiety

This study aimed to examine the impact of the intervention (DTx platform) compared with standard procedural information on children and their parents’ self-reported procedural anxiety when attending a hospital for a planned procedure. Children and parents were asked to rate their procedural anxiety on a single 10-point VAS.

**Figure 3:**
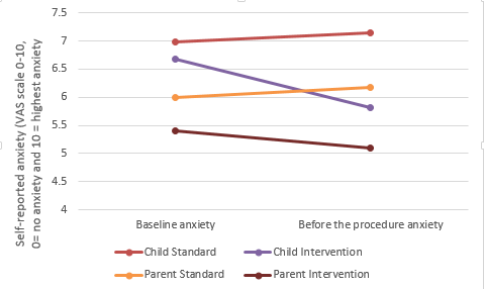
Self-reported procedural anxiety.

At baseline, there were no differences in procedural anxiety between the standard and intervention groups for both parents and children (Table 3). However, before the procedure, the mean procedural anxiety scores of both children and parents in the intervention group were significantly lower than those in the standard group (Figure 3).

**Table 3:** Procedural anxiety levels of children and their parents in the standard and intervention groups.

Timepoint	Children				Parents			
	Standard, mean (SD)	Intervention, mean (SD)	*t* value (*df*)	*P* value	Standard, mean (SD)	Intervention, mean (SD)	*t* value (*df*)	*P* value
Baseline	6.98 (2.70)	6.68 (1.51)	0.613 (78)	.54	6.00 (2.97)	5.40 (2.193)	1.056 (78)	.31
Before procedure	7.15 (2.63)	5.82 (1.57)	2.740 (63.64)	.008	6.18 (2.836)	5.10 (1.919)	1.985 (68.51)	.05

We then conducted two-tailed paired *t* tests to determine whether there were any differences in parents and children’s levels of procedural anxiety from baseline to before the procedure, depending on the nature of the procedure (eg, invasive vs noninvasive procedure). The only statistically significant difference in scores occurred in the intervention group. For children undergoing invasive procedures, levels of anxiety decreased from baseline (mean 6.61, SD 1.50) to before the procedure (mean 5.84, SD 1.54; *t*_30_=2.555; *P*=.02). Similarly, for children undergoing noninvasive procedures, anxiety decreased from baseline (mean 6.89, SD 1.62) to preprocedure (mean 5.78, SD 1.79; *t*_8_=2.857; *P*=.02). For parents, anxiety decreased from baseline (mean 5.71, SD 2.27) to before the procedure (mean 5.32, SD 2.02) for those whose children were undergoing invasive procedures only (*t*_30_=2.834; *P*=.008). The Xploro DTx platform therefore reduced children’s levels of procedural anxiety regardless of the kind of procedure they were undergoing, whereas for parents, the intervention was most beneficial to those whose children were undergoing invasive procedures.

We then examined whether there were any differences in parents and children’s levels of procedural anxiety from baseline to before the procedure, depending on the number of times children reported previous visits to the hospital (eg, less than 3 or more than 3). The only statistically significant difference was found in the intervention group. For those who had visited the hospital 3 times or less, children’s levels of anxiety decreased from baseline (mean 6.77, SD 1.31) to preprocedure (mean 5.54, SD 1.30; *t*_25_=7.273; *P<*.001). The parents of children who had visited the hospital 3 times or less also experienced a reduction in anxiety from baseline (mean 5.54, SD 1.79) to preprocedure (mean 5.15, SD 1.52; *t*_25_=2.813; *P=*.009). Therefore, in terms of anxiety, Xploro benefitted children and parents who had less exposure to hospital environments compared with those who had more exposure.

### Feeling I Know More: Increasing the Perception of Procedural Knowledge

This study aims to explore the impact of using a DTx platform compared with standard procedural information on children and their parents’ self-reported perception of procedural knowledge**.** Self-reported procedural knowledge refers to how much knowledge the child or parent believed they had about the procedure, and this was assessed on a single VAS.

**Figure 4:**
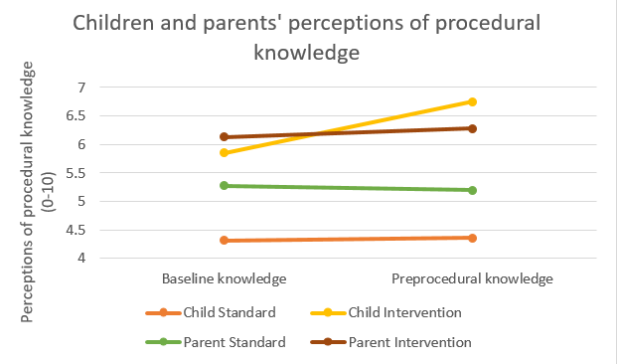
Children and parents' reported perceptions of procedural knowledge.

At baseline, children in the intervention group rated themselves as having significantly higher levels of perceived procedural knowledge compared with children in the control group (Table 4; Figure 4). Similarly, parents perceived procedural knowledge at baseline was higher in the intervention group than in the standard group, although this difference was only marginally significant (Table 4). These findings were unexpected given that the children and parents in the intervention group did not have access to the DTx platform when the baseline measurements were obtained.

**Table 4:** Perceived procedural knowledge of children and their parents in the standard and intervention groups.

Timepoint	Children				Parents				
	Standard, mean (SD)	Intervention, mean (SD)	*t* value (*df*)	*P* value		Standard, mean (SD)	Intervention, mean (SD)	*t* value (*df*)	*P* value
Baseline	4.32 (2.66)	5.85 (1.83)	−2.866 (78)	.006		5.28 (2.29)	6.13 (1.70)	−1.888 (78)	.06
Before procedure	4.36 (2.66)	6.75 (1.51)	−4.892 (59.98)	<.001		5.200 (2.267)	6.28 (1.26)	−2.621 (78)	.01

We then conducted a two-tailed paired *t* test to determine whether there were any differences in parents and children’s levels of perceived procedural knowledge from baseline to before the procedure, depending on the nature of the procedure (eg, invasive procedure vs noninvasive procedure). The only statistically significant difference in scores occurred in the intervention group. For children undergoing an invasive procedure, there was a statistically significant increase in perceptions of procedural knowledge from baseline (mean 6.10, SD 1.81) to before the procedure (mean 6.78, SD 1.38; *t*_30_=−3.760; *P*=.001). Similarly, for children undergoing noninvasive procedures, there was a statistically significant increase in perceptions of procedural knowledge from baseline (mean 5.00, SD 1.73) to before the procedure (mean 6.33, SD 1.94; *t*_8_=−2.412; *P*=.04). Xploro was therefore of the greatest benefit to children, regardless of the kind of procedure under.

We then examined whether there were any differences in parents and children’s levels of perceptions of procedural knowledge from baseline to before the procedure, depending on the number of times the children had visited the hospital (eg, less than 3 or more than 3). The only statistically significant difference was found in the intervention group. For children who had fewer than 3 visits, there was a statistically significant increase in perceived procedural knowledge from baseline (mean 5.69, SD 1.81) to before the procedure (mean 6.54, SD 1.45; *t*_25_=−3.528; *P*=.002). Similarly, for those who had 3 or more visits, there was a statistically significant increase in perceived procedural knowledge from baseline (mean 6.14, SD 1.92) to preprocedure (mean 7.14, SD 1.61; *t*_13_=−2.646; *P*=.02). In terms of procedure knowledge, the Xploro DTx platform therefore benefitted children and parents perceived procedural knowledge regardless of their previous exposure to a hospital.

### Feeling Happier About What Happened: Procedural Satisfaction

Children and their parents’ self-reported satisfaction of undergoing the hospital procedure was rated after the procedure on a single 10-point VAS. Both parents and children’s procedural satisfaction scores were higher in the intervention group compared with the standard group, although these differences were not statistically significant (Table 5).

**Table 5:** Procedural satisfaction of children and their parents in the standard and intervention groups.

Timepoint	Children				Parents			
	Standard, mean (SD)	Intervention, mean (SD)	*t* value (*df*)	*P* value	Standard, mean (SD)	Intervention, mean (SD)	*t* value (*df*)	*P* value
After the procedure	5.98 (2.787)	6.88 (2.002)	-1.659 (70.79)	.10	6.63 (2.457)	6.80 (1.800)	-0.363 (78)	.72

### Feeling More Involved: Increasing Procedural Involvement

The study aimed to understand whether children and parents who received the intervention would report higher levels of procedural involvement. Children were asked after the procedure to rate their perceived levels of involvement on a 3-point Likert scale. After the procedure, the mean procedural involvement scores of children in the intervention group were significantly higher than those in the standard group (*t*_75_=−2.238; *P*=.03).

### Using It and Liking It: Children’s Engagement With the Intervention

This study also aimed to understand the levels of engagement a child had with the intervention (DTx platform). Although software was not used to determine clear statistical levels of engagement, the questionnaire booklets asked the children to retrospectively record their levels of engagement with the intervention. This enabled us to gain some understanding of which parts of the DTx platform were used. All of the children who accessed and used the intervention valued the content and enjoyed the various components, particularly the customized avatar and chatbot. Table 6 identifies the specific components of the intervention that the children used.

The children were asked to rate how much they liked the different components of Xploro that they used. Only 20 out of the 40 children in the intervention group completed this section, but those who did reported that they liked all the different components they accessed.

**Table 6:** Children’s engagement with the different components of the intervention (n=40).

Different components of the intervention			Children reporting using each component, n (%)
Avatar			40 (100)
Chatbot			40 (100)
Ward orientation			36 (90)
**Equipments**			
	MRI^a^	22 (55)	
	Monitor	24 (60)	
	X-ray machine	24 (60)	
	LINAC^b^	24 (60)	
	CT^c^ scan	24 (60)	
	Ultrasound	4 (10)	
Who is who (staff)			28 (70)
**Games**			
	Heart race	28 (70)	
	Germ buster	28 (70)	
	And relax	26 (65)	
	Operating room	30 (75)	
	Anesthesia room	30 (75)	
	Ward	39 (98)	
	Recovery room	25 (63)	

^a^MRI: magnetic resonance imaging.

^b^LINAC: linear particle accelerator.

^c^CT: computed tomography.

### Qualitative Experiences of Using the Intervention

#### It Helped Me to Understand What Would Happen: Experiences of Children Using the Intervention

During the semistructured *write and tell* interviews, the children stated that they enjoyed using the intervention and felt that it had positively influenced their experiences of coming to the hospital for a procedure. The interviews demonstrated that the intervention benefitted the children by providing them with information and knowledge about the procedure they were about to undergo while at home “it is so good, like being to see the hospital while you are still at home” (P24). One child stated that the DTx platform helped them “know things I didn’t know before, I didn’t know all the tests would be from one needle I thought it was loads” (P12), while another talked about how the intervention “is great, it taught me lots and I knew what all the numbers on the machine were and made me feel less worried” (P3). Even children who had previous experience with a procedure valued the information: “It [Xploro] is good, I have had an MRI before but I didn’t know what it was doing while I was in it–but now I know” (P1).

The children also reported using the intervention to distract themselves and take their minds off their procedure. This was particularly linked to the games that were viewed positively by the children: “they were fun and helped to distract me” (P37); “I liked playing the games on the iPad” (P36); “it helped when I was having my stitches out as I could play on it and not think about it” (P21).

The interviews also helped to identify the important roles that parents play in facilitating or disabling children’s access to information: “my mum only let me play on it last night” (P20); “if it was a game in real life I would ask my mum for it” (P14).

#### It Helped Us Talk About What Would Happen: Experiences of Parents of Their Child Using the Intervention

The parents discussed how their child had enjoyed using Xploro and how the intervention had helped provide children with information about the procedure: “it helped him learn lots about the scan” (P13) and “it [Xploro] is great, she now knows more than me” (P3); parents described how without the intervention their child may have been lacking in information “the nurse didn’t explain so without it [Xploro] she wouldn’t have had a clue” (P19). The increase in knowledge was reported by parents as reducing their child’s anxiety “she feels more relaxed as she knows what things look like and knows about the anaesthetic machine” (P39).

Parents also reported that the intervention helped them talk about the planned procedure with their child, “playing with the app allowed us to talk about what would happen, otherwise I wouldn’t know how to approach it” (P1). Despite the intervention being focused on providing information to children, some parents found it really useful to them, “It [Xploro] is amazing, I used it more than him” (P7), although some parents did not appreciate that the focus of the intervention was on information provision as well as being fun, “if I had known it was about learning and not just games I would have let her use it sooner” (P20). The engaging components were viewed positively by parents, “she liked the interactive bits, it looks good” (P37) and “he loved making the man” (P7).

## Discussion

### Principal Findings

We report the results of a before and after evaluation study to examine the acceptability of a DTx platform (Xploro) and the impact of this intervention on children undergoing a clinical procedure and their parents. To the best of our knowledge, this is the first study to evaluate the impact of a DTx platform using gamification, serious games, a chatbot, and an augmented reality avatar with children undergoing a wide range of hospital procedures. This study provides preliminary evidence that the intervention (Xploro) reduced procedural anxiety of children and their parents and improved children’s perceptions of procedural knowledge and involvement. All the children who accessed and used the DTx platform described how they valued the content, enjoyed using the various components, and reported that it improved their procedural experience by helping them be more prepared, less anxious, and more distracted. These findings suggest that the Xploro intervention has the potential to improve children’s procedural health literacy and address the need for meaningful and accessible procedural information. This is the first study to focus on the influence of a digital health intervention on children’s procedural health literacy. To our knowledge, this is the first study to evaluate a DTx platform by measuring multiple concepts including children’s procedural anxiety, perceptions of knowledge, procedural satisfaction, and perceived procedural involvement.

The DTx platform provides information about a broad range of hospital procedures, environments, and professionals; this is important as children can often encounter many different experiences when visiting the hospital, which may not be addressed by current digital health education and preparation interventions that focus on one specific procedure, for example, admission for surgery [13-16,29], or are designed around one specific hospital setting [30]. These interventions are of value but may not address the questions and concerns children have about procedures identified in previous research [9].

Children particularly valued that the Xploro intervention enabled them to customize and individualize their learning experiences; they could access different components of Xploro based on their individual preferences and information needs. Previous research indicates that children wish to receive information specific to them and are more likely to engage with and understand information delivered in a child-centered way [10]. It is increasingly recognized within the literature, particularly in relation to children, that health literacy is facilitated when an individual can tailor information to their needs and have the opportunity to process, question, and apply information to their individual circumstances [9] and actively construct knowledge and understanding [31]. This tailoring and individualization of information takes time, and interventions need to assist and support children in doing this. The Xploro intervention, underpinned by an *information-pull* approach, facilitated children to determine and address their own information needs and actively engage with the multiple elements of the intervention to shape their own learning experience. Digital health interventions that children can use flexibly and actively to pace their own learning are potentially of most value, based on evidence showing how children require different procedural information and education depending on their cognitive development and a previous experience [32]. Studies exploring the use of virtual reality interventions have also highlighted the importance of active user engagement and how passive interaction with an intervention is linked to poorer impact and experience [33].

There is an increasing need for high-quality and evidence-based digital solutions to the National Health Service’s challenges [34]. This is against the backdrop of the need for improved innovation and implementation [24] to meet these health care challenges. The team are committed to developing a digital health intervention for children, which has been rigorously developed and evaluated based on a child-centered approach. This study reports on the findings of a before and after study, which provides evidence that a larger effectiveness study would be of value.

### Strengths and Limitations

One of the main strengths of the study were the methods used to ensure children’s views and self-reported experiences were central to the investigation. Further strengths are the similarities in the demographic characteristics between the intervention and standard care groups and the demonstrated suitability of the recruitment and data collection processes.

A potential limitation is that the study participants were not randomly allocated to the different groups, and the study was conducted in one hospital setting. The analyses presented are exploratory in nature. Given the number of statistical tests that have been conducted and the relatively modest sample size, generalizations should be made with caution. The study only used single-item scales to measure the concepts of procedural anxiety, perception of knowledge, satisfaction, and procedural involvement; the use of multi-item scales may have provided more robust data. Future studies should validate these preliminary findings in a larger controlled study, which also examines the implementation of the intervention within clinical services.

### Conclusions

Large numbers of children undergo clinical procedures every day within a hospital setting. Many of these children are currently not well-prepared or well-informed about what will happen, which can lead to high levels of procedural anxiety and distress. This study has shown that the Xploro DTx platform improved children’s reported procedural involvement and perceived procedural knowledge. The intervention reduced both parents and children’s levels of procedural anxiety before the procedure. The children and parents in the intervention group described Xploro as easy to use and fun, which helped them to know more before the planned procedure and how to cope during the procedure.

## Abbreviations

DTx: digital therapeutic
VAS: visual analog scale
